# Supplementary material for: Phase I Study of a B Cell-Based and Monocyte-Based Immunotherapeutic Vaccine, BVAC-C in Human Papillomavirus Type 16- or 18-Positive Recurrent Cervical Cancer
Source: J Clin Med. 2020 Jan 5;9(1):147. doi: 10.3390/jcm9010147 (PMC7019768; doi:10.3390/jcm9010147)
Supplement: Supplementary file 1 [file jcm-09-00147-s001.pdf]

Supplemental Material

Table 1. Scheduled visits for treatment, clinical and immunological assessment in the BVAC-C phase I trial.

Study timeline	Screening	Visit 1	Visit 2		Visit 3	Visit 4		Visit 5	Visit 6		Visit 7	Visit F
	-6 w<br>~-1 w	-2 w<br>~4 d	0 w<br>±2 d	0 w<br>+1 d	2 w<br>±2 d	4 w<br>±2 d	4 w<br>+1 d	6 w<br>±2 d	8 w<br>±2 d	8 w<br>+1 d	10 w<br>±2 d	12 w<br>±2 d
Enrollment	●											
Lymphapheresis		●										
BVAC-C injection			●			●			●			
AE assessment	●	●	●	●	●	●	●	●	●	●	●	●
Serum cytokine assay			●	●			●	●		●	●	
CD4/CD8 T cell assay	●				●	●		●	●		●	●
Radiological assessment	●											●

Abbreviations: AE = Adverse Event; w = week; d = day; F = final

Table 2. Adverse events of any grade observed in the study (*n* = 11).

Adverse events	$1x10^7$ (*n* = 4)			$4x10^7$ (*n* = 3)			$1x10^8$ (*n* = 4)			Total (*n* = 11)		
	**G1**	**G2**	**G3**	**G1**	**G2**	**G3**	**G1**	**G2**	**G3**	**G1 (%)**	**G2 (%)**	**G3 (%)**
Respiratory, thoracic and mediastinal disorders												
Oropharyngeal pain	1	0	0	0	0	0	0	0	0	**1 (9)**	**0**	**0**
Cough	0	0	0	1	0	0	0	0	0	**1 (9)**	**0**	**0**
Productive cough	0	0	0	1	0	0	0	0	0	**1 (9)**	**0**	**0**
Dyspnea	0	0	0	1	0	0	0	0	0	**1 (9)**	**0**	**0**
Nervous system disorders												
Headache	1	0	0	1	0	0	1	0	0	**3 (27)**	**0**	**0**
Musculoskeletal and connective tissue disorders												
Myalgia	2	1	0	1	0	0	2	0	0	**5 (45)**	**1 (9)**	**0**
Pain in extremity	0	0	1	0	0	0	0	0	0	**0**	**0**	**1 (9)**
Bone pain	1	0	0	0	0	0	0	0	0	**1 (9)**	**0**	**0**
Back pain	0	0	0	0	0	0	0	1	0	**0**	**1 (9)**	**0**
Musculoskeletal pain	0	0	0	0	0	0	0	1	0	**0**	**1 (9)**	**0**
General disorders and administration site conditions												
Flank pain	1	0	0	0	0	0	0	0	0	**1 (9)**	**0**	**0**
Pyrexia	1	0	0	3	0	0	3	0	0	**7 (63)**	**0**	**0**
Asthenia	0	0	0	2	0	1	0	0	0	**2 (18)**	**0**	**1 (9)**
Edema peripheral	0	0	0	1	0	0	0	0	0	**1 (9)**	**0**	**0**
Chills	0	0	0	0	0	0	2	0	0	**2 (18)**	**0**	**0**
Fatigue	0	0	0	0	0	0	1	0	0	**1 (9)**	**0**	**0**

Gastrointestinal disorders												
Constipation	1	0	0	0	0	0	1	0	0	**2 (18)**	**0**	**0**
Dyspepsia	0	0	0	1	0	0	1	0	0	**2 (18)**	**0**	**0**
Abdominal distension	0	0	0	1	1	0	0	0	0	**1 (9)**	**1 (9)**	**0**
Ileus	0	0	0	1	0	1	0	0	0	**1 (9)**	**0**	**1 (9)**
Nausea	0	0	0	1	0	0	0	0	0	**1 (9)**	**0**	**0**
Stomatitis	0	0	0	1	1	0	0	0	0	**1 (9)**	**1 (9)**	**0**
Diarrhea	0	0	0	0	0	0	0	1	0	**0**	**1 (9)**	**0**
Vomiting	0	0	0	0	0	0	1	0	0	**1 (9)**	**0**	**0**
Metabolism and nutrition disorders												
Decreased appetite	0	1	0	0	0	0	0	0	0	**0**	**1 (9)**	**0**
Hyperkalemia	0	0	0	0	1	0	0	0	0	**0**	**1 (9)**	**0**
Hypoalbuminemia	0	0	0	0	2	1	0	0	0	**0**	**2 (18)**	**1 (9)**
Hypophagia	0	0	0	2	0	1	0	0	0	**2 (18)**	**0**	**1 (9)**
Neoplasms benign, malignant and unspecified												
Skin papilloma	0	1	0	0	0	0	0	0	0	**0**	**1 (9)**	**0**
Blood and lymphatic system disorders												
Anemia	0	0	0	1	1	1	1	2	1	**2 (18)**	**3 (27)**	**2 (18)**
Hepatobiliary disorders												
Ascites	0	0	0	0	0	1	0	0	0	**0**	**0**	**1 (9)**
Renal and urinary disorders												
Bladder infections and inflammation	0	0	0	1	0	0	0	0	0	**1 (9)**	**0**	**0**
Nocturia	0	0	0	0	1	0	1	0	0	**1 (9)**	**1 (9)**	**0**
Ureteric stenosis	0	0	0	1	0	0	0	0	0	**1 (9)**	**0**	**0**
Incontinence	0	0	0	0	0	0	1	0	0	**1 (9)**	**0**	**0**

Investigations												
C-reactive protein increased	0	0	0	0	0	0	1	0	0	**1 (9)**	**0**	**0**
Immune system disorders												
Cytokine release syndrome	0	0	0	0	0	0	0	1	0	**0**	**1 (9)**	**0**
Injury, poisoning and procedural complications												
Ligament sprain	0	0	0	0	0	0	1	0	0	**1 (9)**	**0**	**0**
Reproductive system and breast disorders												
Pelvic inflammatory disease	0	0	0	0	0	0	0	1	1	**0**	**1 (9)**	**1 (9)**
